# PERSONALIZED AND TYPICAL CONCURRENT RISK: JOINT MODELS OF RECURRENT OUTCOMES AND MORTALITY BY RACE/ETHNICITY

**DOI:** 10.1093/geroni/igad104.0759

**Published:** 2023-12-21

**Authors:** Heather Allore, Gail McAvay, Brent Vander Wyk, Ling Han, Ana Quiñones

**Affiliations:** Yale University, New Haven, Connecticut, United States; Yale University, New Haven, Connecticut, United States; Yale University, New Haven, Connecticut, United States; Yale School of Medicine, New Haven, Connecticut, United States; Oregon Health and Science University, Portland, Oregon, United States

## Abstract

The growing burden of multiple chronic conditions documented by a U.S. Department of Health and Human Services initiative noted the importance of studies that 1) “Develop tools to identify and target population subgroups of individuals with multiple chronic conditions who are at high risk for poor health outcomes”, and 2) “Improve knowledge about patient trajectories temporally in relation to changes in health status, functional status, and health services use”. Expanding upon our methods for jointly modeling multiple outcomes, where each person’s shared influence on concurrent outcomes can be captured by using a random intercept term, we demonstrate how to model additional heterogeneity. Based on the estimates from the joint model, the typical concurrent risk (TCR) of each outcome (i.e., probability) can be estimated at the cohort level, providing a method for identifying average longitudinal effects of risk factors useful for healthcare system policies. In contrast, a person-specific effect reflects the probability of each outcome within a defined interval of time, while currently at risk for another non-mutually exclusive outcome (i.e., personalized concurrent risk [PCR]). Chronic conditions and sociodemographic risk factors may partially account for the heterogeneity of a cohort. However, differences in the occurrences and associations among these may differ for minoritized groups. These methods identify persons above (or below) average risk. We expanded these joint models to address truncation of measurement due to death and the interdependence of outcomes at the person-level, while maintaining the Type I error rate. We have made the code publicly available.

